# Clinical advantages of gradually reducing radius versus multi-radius total knee arthroplasty: a noninferiority randomized trial

**DOI:** 10.1186/s12891-023-06177-4

**Published:** 2023-01-26

**Authors:** Sakkadech Limmahakhun, Anuchit Chaiamporn, Kasisin Klunklin, Warakorn Jingjit

**Affiliations:** Department of Orthopedic Surgery, Faculty of Medicine, Chiang Mai University, Chiang Mai, Thailand

**Keywords:** Gradually reducing femoral component, Multi-radius femoral component, Total knee arthroplasty

## Abstract

**Background:**

The rationale for gradually reducing radius (GR) femoral component aims to prevent flexion instability by gradually change the center of femoral rotation, unlike a discrete change by the multi-radius (MR) which is more common for most of total knee arthroplasties (TKA). However, no strong evidence has been reported the clinical significance of the GR design.

**Methods:**

This patient-blinded, parallel, non-inferiority trial conducted in September 2020. Patients with knee osteoarthritis consented for cruciate retaining TKA were randomly allocated to a GR or MR group. Primary outcome measures were knee functions at postoperative 6 and 12 months using the Knee injury and Osteoarthritis Outcome Score (KOOS). Secondary outcome measures were performance-based tests (30-s chair stand test, 40-m fast paced walk test, and 3-m timed up and go test), and knee motions.

**Results:**

Sixty patients were enrolled and randomized; GR (*n* = 30) and MR (*n* = 30) group. The changes of KOOS at 6 and 12 months from baseline showed clinical meaningful for both GR and MR group. At 6 and 12 months postoperatively, there was no significant difference between both groups in all KOOS subscales. The length of stay was not different between GR and MR group (5.93 ± 1.44 vs 6.17 ± 1.86 days, *p* = 0.59). Patients on both groups presented similar performance-based tests. However, the improvement in degrees of knee motion for the GR group was significantly greater than the MR group (34.67 ± 12.52 vs 23.67 ± 12.59, *p* = 0.001).

**Conclusion:**

GR was noninferiority to MR for the functional outcomes and performances after TKA.

The GR femoral component gave more knee motions than did the MR prostheses.

**Level of evidence:**

Level I, therapeutic study.

## Introduction

Total knee arthroplasty (TKA) is a very successful operation with an excellent outcome and survivorship results. Patient satisfaction is one of the important outcome measurements because it reflects the overall pain relief and an ability to return to normal activities of daily living after knee replacements. However, only approximately 75% of patients who had a knee replacement satisfied with the total knee arthroplasty (TKA) [1, 2]. Pain relief and knee functional gain after the TKA are sometimes overestimated and lead to patient’s unsatisfaction with the TKA [2]. Filling this gap interested clinicians over the last decades to improve surgical techniques such as less invasive surgery, tourniquet less technique, use of tranexamic acid for perioperative blood management, and periarticular injection; however, pain reduction is promising only in an acute postoperative period.

Not only the surgical technique but also an innovative implant development might be the factors increasing the outcome [3]. The evolution of prosthesis design was developed in order to gain normal knee functions. Frankle et al. [4] proposed the native knee flexion occurring around a changing transvers axis, with the instantaneous rotation center of the femoral posterior condyle forming as a “J curve” [4, 5]. The multi-radius (MR) femoral component was then theoretically invented by using the trans-epicondylar axis as a reference axis of knee flexion and rotation. A different radius of which a larger for a distal and a smaller for a posterior femoral component on the sagittal view aims to allow more degrees of freedom on knee flexion by posterior femoral roll-back and rotation [6]. The conventional MR TKA is the most commonly used design such as PFC Sigma, Depuy Synthes, Warsaw, Indiana; and Nexgen, Zimmer Biomet, Warsaw, Indiana. However, abrupt reduction between the discrete distal and posterior radii for MR TKA causes abrupt changes in the center of rotation of the femur with respect to the tibia and abrupt reductions in tibiofemoral conformity, resulted in a decrease in anterior–posterior tibiofemoral stability and mid flexion instability [7].

**Fig. 1:**
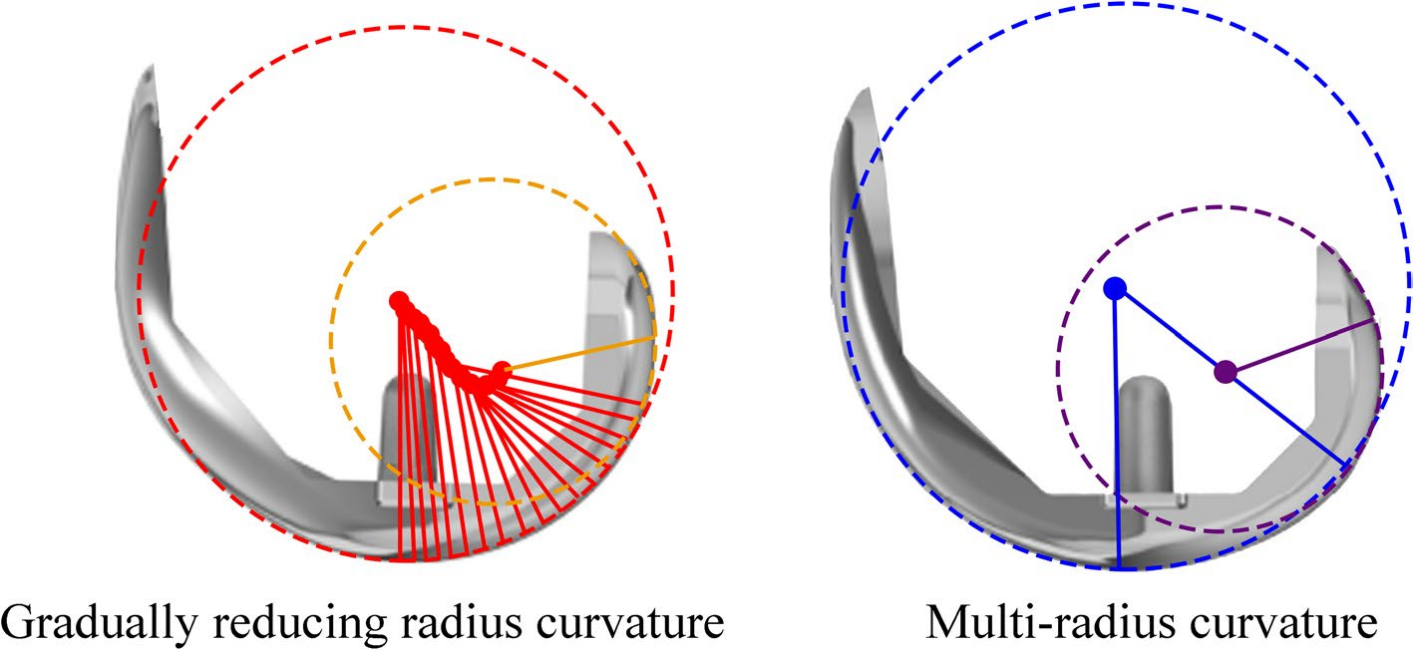
Showed the sagittal femoral component between gradually reducing radius (GR) and multi-radius (MR) design

In 2013, Depuy Synthese launched the gradually reducing radius (GR) design (ATTUNE, Depuy Synthes, Warsaw, Indiana). The GR design allows smooth transition from knee extension to flexion by create a point of rotation similar to the “J curve” in the native knee, Fig. 1. The design rationale aimed to prevent the flexion instability by gradually change the center of femoral rotation to produce a more proper ligament tension throughout the arch of knee motion, whereas the posterior femoral roll-back was still preserved unlike the single-radius design [6].

To increase the TKA outcome, modern knee prostheses are designed to match a native knee geometry, which anticipated to mimic the knee kinematic close to a normal knee [7, 8]. While, the strongest predictor of patient dissatisfaction after primary TKA was expectations not met [2], lack of clinical evidence for a GR modern knee prosthesis to inform patients preoperatively was a challenging. Therefore, this prospective study aims to explore any clinical significance between the gradually radius (GR) femoral component versus multi-radius (MR) femoral component in primary TKA.

## Materials and methods

### Trial design

**Fig. 2:**
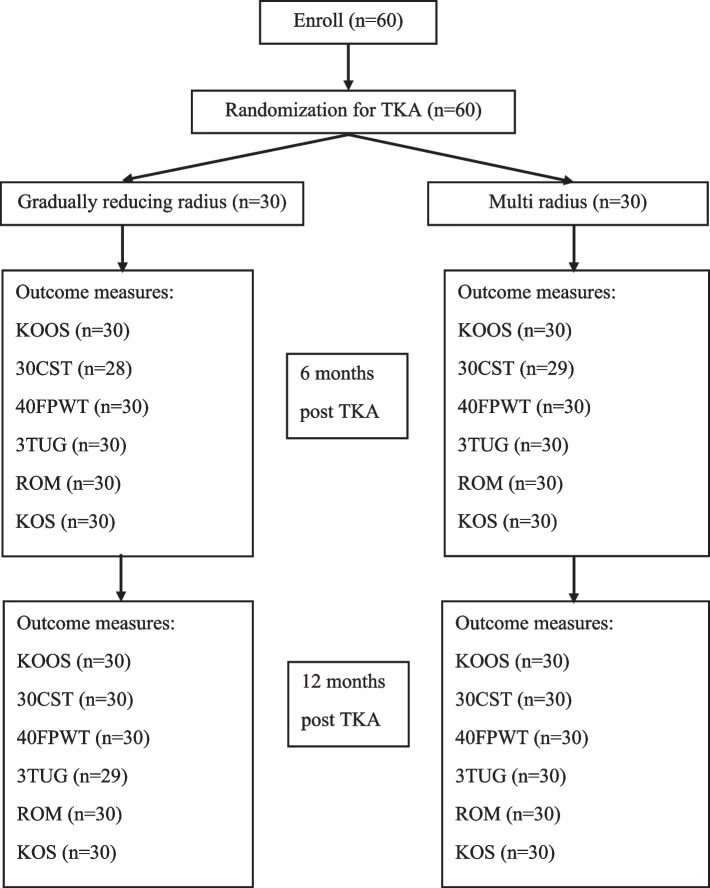
CONSORT flow diagram depicting participant flow throughout the study, from eligibility through enrollment, intervention, and data collection

The trial was a single-center, CONSORT-compliant, patient-blinded, 2-group, parallel randomized controlled trial (RCT) conducted in September 2020 as demonstrated in Fig. 2. The trial was prospectively approved by the Institutional Ethical Board Committee (ORT-2563–07,318); however, retrospective registration was approved on 30/10/2022, TCTR20221030001, due to COVID situation and human error. All participants gave written informed consent to the study inclusion and randomization before the enrollment.

### Setting and recruitment

Participants were recruited from an orthopedic outpatient clinic at Maharaj Nakorn Chiang Mai Hospital, Thailand, between September 2020 and August 2021. Patients were approached by the study coordinator during their attendance at the clinic following surgeon assessment for TKA and assessed for eligibility and willingness to participate. Eligible criteria included patients 50–80 years of age with a clinical and radiographic diagnosis of severe knee osteoarthritis (Kellgren and Lawrence grade IV) by one of the arthroplasty consultants and placed on the waiting list for unilateral TKA. Exclusion criteria were revision surgery, history of septic or inflammatory arthritis, body mass index (BMI) > 36 kg/m^2^, severe coronal deformity > 20° which would be potentially deleterious to postoperative outcome if the patient was randomized, and unable to ambulate independently preoperatively.

### Randomization and blinding

Participants were randomly assigned into one of the two groups either GR or MR with a ratio of 1:1 using a computerized stratified block randomization sequence (block of 4). When a subject fulfilled all study criteria and was enrolled, allocation took place. Allocation concealment was performed using sequentially numbered, opaque, sealed envelopes (SNOSE) prepared by the independent person that was not relevant to this study. Participants also remained blinded to the type of prosthesis throughout the study. The two surgeons performing TKA were informed of participant assignment 1 month prior to surgery and had no role in patient assessment. The blinded study coordinator was accountable for gathering of outcome measures.

### Interventions

All participants underwent TKA and postoperative care following the hospital’s routine TKA program, which has been standardized through the use of clinical pathway protocols. Demographic data of patients including age, sex, body mass index, length of stay, pre-operative range of motion and ASA were collected. All TKA designs were performed using a cruciate retaining (CR) prosthesis from two manufacturers; ATTUNE (Depuy Synthes, Warsaw, Indiana) as a GR group and LEGION (Smith & Nephew, Memphis, TN) as a MR group. Patients receive a spinal anesthesia with peripheral nerve block prior to surgery. All cemented TKAs were performed with the same technique by the same experience surgeons; SL and KK, under tourniquet control. A standard midline skin incision was used, and the arthrotomy was done using a medial parapatellar approach. The distal femoral cut was made with a valgus angle of 6° to the femur anatomical axis using an intramedullary cutting guide, while the proximal tibial cut was made perpendicular to the mechanical axis using an extramedullary guide. Femoral sizing was measured using femoral sizing jig with the anterior referencing technique before anterior, posterior, and chamfer bony cuts were completely removed. Femoral rotational alignment was performed according to the epicondylar axis, usually 3° of external rotation from the posterior condylar line. Posterior femoral osteophyte was routinely removed before the tibial surface preparation. A range of 7°-10° of posterior tibial slope was aimed along with the native tibial slope. Patella was selective resurfaced only patella mal-tracking and/or severe patellofemoral joint arthritis was found. All patients were scheduled for the 6- and 12-months follow-up with the study coordinator at the same clinic for clinical review.

### Primary outcome

The primary outcome was the knee function at postoperative 6 and 12 months using the Knee injury and Osteoarthritis Outcome Score (KOOS) and the performance-based tests. The KOOS is a patient-reported outcome questionnaire to evaluate pain, symptom, activity daily living, sport, and quality of life after TKA. The performance-based tests used in this study were 30-s chair stand test (30CST), 40-m fast paced walk test (40FPWT), and 3-m timed up and go test (3TUG).

### Secondary outcome

The secondary outcome was length of stay and range of motion at 6 and 12 months postoperatively. Pre and postoperative knee alignments were determined using hip knee axis (HKA) on weight-bearing scanogram. The component positions were evaluated by the mechanical lateral distal femoral angle (mLDFA), mechanical medial proximal tibial angle (mMPTA), and tibial slope angle (TSA).

### Statistical analysis

Prosthesis performance was evaluated by comparing change in the primary and secondary outcomes between two groups using SPSS 23.0 (IBM) at a level of significance of 0.05. We aimed to detect the minimum clinically important difference in the KOOS between groups, which the noninferiority margin was set at 15.1 points based on the previous study by Hung et al. [9]. The sample size calculation was based on an inferiority test with continuous outcome using a covariance adjusting for baseline scores, estimating between-patient standard deviations of 18.5 points [10], an alpha value = 0.05, and 2-sided test and power = 80%. A total of 20 participants per each group were required. We aimed to recruit 60 participants to allow for a 20% drop-out rate. This trial can end when the sample size and follow-up goal were reached. Continuous variables were assessed by independent t tests. Descriptive statistics are displayed as means with standard deviation or range for continuous variables.

## Results

A total of 60 patients (male 20 and female 40) were enrolled and randomized for the TKA allocation; which was 30 patients in both GR and MR groups. No participant withdrawn during the follow-up. Data was completely collected in August 2022. At 6 months post TKA, three participants were unable to performed the 30CST due to dizziness, and at 12 months post TKA, one participant was unable to perform 3TUG test due to palpitation. Average patient age of GR group and MR group were 67 (59–82) and 69 (61–86) years, respectively. The characteristics of each group were similar at baseline (Table 1). The length of stay was not different between GR and MR group (5.93 ± 1.44 days vs 6.17 ± 1.86 days, *p* = 0.59).

**Table 1:** shows demographic data between gradually reducing radius (GR) versus multi-radius (MR) TKA groups

	GR TKA (*n* = 30)	MR TKA (*n* = 30)	*P*-value
Age (y)	67 (59–82)	69 (61–86)	
Gender			
Male	9 (30%)	11 (36.67%)	
Female	21 (70%)	19 (63.33%)	
Body mass index (kg/m^2^)	25.76 ± 3.47	26.33 ± 3.74	
Preop ROM			
Extension (degrees)	9.83 ± 4.64	7.5 ± 5.37	
Flexion (degrees)	77.17 ± 8.48	82.67 ± 8.78	
Range of motion (degrees)	67.33 ± 10.56	75.17 ± 12.76	
ASA			
1	1 (3.33%)	1 (3.33%)	
2	27 (90%)	27 (90%)	
3	2 (6.67%)	2 (6.67%)	
4	0	0	
Radiographic evaluation			
Preop HKA (95% CI)	168 ± 5 (164–171)	168 ± 6 (164–171)	0.8
Postop HKA (95% CI)	176 ± 3 (174–177)	175 ± 3 (173–177)	0.38
mLDFA (95% CI)	91 ± 3 (89–93)	91 ± 3 (88–93)	0.8
mMPTA (95% CI)	88 ± 2 (87–90)	87 ± 2 (86–88)	0.1
TSA (95% CI)	7 ± 1 (6–8)	7 ± 1 (6–8)	0.77

### Outcomes

**Fig. 3:**
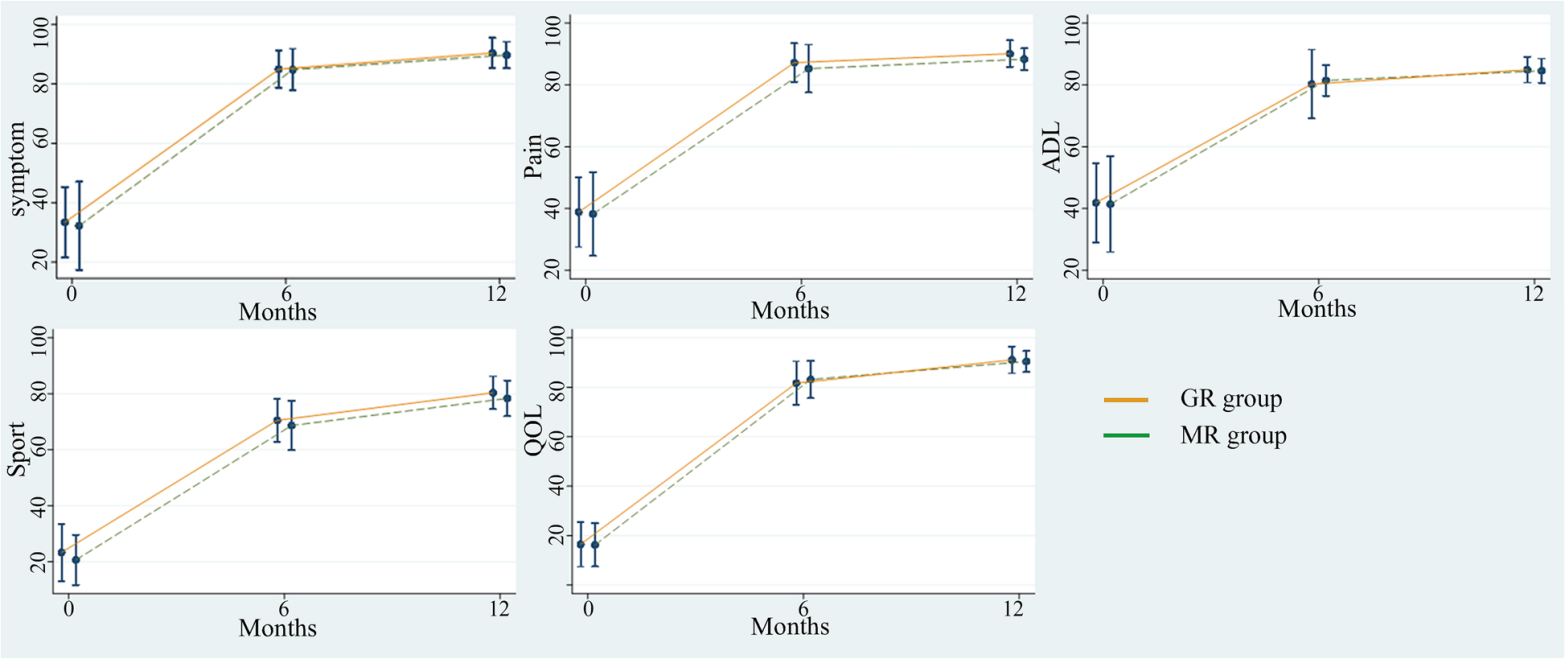
Showed KOOS score in symptom, pain, activities of daily living (ADL), sport and recreation function, and quality of life (QOL) between gradually reducing radius (GR) vs multi-radius (MR) femoral component TKA

At 6 months and 12 months postoperatively, there was no significant difference between GR and MR group in all subscales of KOOS, Table 2. The changes of KOOS at 6 and 12 months from baseline showed clinical meaningful for both GR and MR group, Fig. 3. However, no statistical difference of the magnitude of KOOS change between 6 and 12 months from baseline was found between each group, Table 3. Performance-based tests were assessed from pre-operation and at 6 and 12 months post TKA. There was no significant difference between GR and MR group for 30CST, 40FPWT, and 3TUG, Table 4.

**Table 2:** showed the KOOS at baseline, 6 month and 12 months postoperatively

KOOS score	GR design (95% CI)	MR design (95% CI)	*P*-Value
Symptom			
Preoperative	33.43 ± 11.87 (29–37.9)	32.26 ± 14.90 (27–37.8)	0.73
Postoperative 6 months	84.96 ± 6.24 (82.6–87.3)	84.82 ± 7.01 (82.2–87.4)	0.93
Postoperative 12 months	90.39 ± 5.14 (88.5–92.3)	89.76 ± 4.47 (88.1–91.4)	0.61
Pain			
Preoperative	38.84 ± 11.28 (34.6–43.1)	38.23 ± 13.53 (33.2–43.3)	0.85
Postoperative 6 months	87.21 ± 6.28 (84.9–89.5)	85.28 ± 7.73 (82.4–88.2)	0.29
Postoperative 12 months	90.09 ± 4.29 (88.5–91.7)	88.33 ± 3.53 (87–89.6)	0.09
Activities of daily living			
Preoperative	41.89 ± 12.85 (37.1–46.7)	41.42 ± 15.50 (35.6–47.2)	0.89
Postoperative 6 months	80.24 ± 11.14 (76.1–84.4)	81.42 ± 4.98 (79.6–83.3)	0.59
Postoperative 12 months	84.90 ± 4.18 (83.3–86.5)	84.56 ± 3.91 (83.1–86)	0.74
Sport/Recreation			
Preoperative	23.33 ± 10.20 (19.5–27.1)	20.67 ± 8.98 (17.3–24)	0.28
Postoperative 6 months	70.5 ± 7.70 (67.6–73.4)	68.67 ± 8.80 (65.4–71.9)	0.39
Postoperative 12 months	80.33 ± 5.86 (78.1–82.5)	78.33 ± 6.34 (76–80.7)	0.21
Quality of life			
Preoperative	16.46 ± 9.06 (13.2–19.5)	16.25 ± 8.78 (14.1–18.6)	0.85
Postoperative 6 months	81.67 ± 8.83 (78.4–85)	83.13 ± 7.55 (80.3–86)	0.49
Postoperative 12 months	91.04 ± 5.36 (89–93)	90.42 ± 4.26 (88–92)	0.62

**Table 3:** showed mean change in KOOS scores between GR and MR group at 6 and 12 months from baseline

Mean change in KOOS score	GR TKAs (95%CI)	MR TKAs (95%CI)	*P*-Value
Symptom			
Preoperative – Postoperative 6 months	51.53 ± 13.50 (46.48–56.57)	52.56 ± 14.88 (47.00–58.11)	0.78
Preoperative – Postoperative 12 months	56.96 ± 11.12 (52.8–61.1)	57.50 ± 115.84 (51.58–63.41)	0.88
Pain			
Preoperative – Postoperative 6 months	48.37 ± 13.67 (43.26–53.47)	47.05 ± 15.45 (41.27–52.81)	0.73
Preoperative – Postoperative 12 months	51.25 ± 12.04 (46.75–55.74)	50.10 ± 13.54 (45.0–55.15)	0.73
Activities of daily living			
Preoperative – Postoperative 6 months	38.36 ± 14.39 (32.98–43.73)	40.00 ± 15.88 (34.07–45.93)	0.67
Preoperative – Postoperative 12 months	43.01 ± 13.50 (37.96–48.05)	43.14 ± 15.20 (37.46–48.81)	0.97
Sport/Recreation			
Preoperative – Postoperative 6 months	47.17 ± 13.04 (42.29–52.04)	48 ± 11.72 (43.62–52.37)	0.79
Preoperative – Postoperative 12 months	57 ± 11.64 (52.65–61.35)	57.67 ± 8.68 (54.42–60.90)	0.80
Quality of life			
Preoperative – Postoperative 6 months	65.21 ± 11.69 (60.84–69.57)	66.88 ± 12.19 (62.32–71.42)	0.59
Preoperative – Postoperative 12 months	74.58 ± 10.37 (70.71–78.45)	74.17 ± 9.10 (70.77–77.56)	0.87

**Table 4:** showed performance-based tests from preoperative TKA, 6 months post TKA, and 12 months post TKA

Performance-based tests	GR TKAs (95%CI)	MR TKAs (95%CI)	*P*-Value
30 s chair stand test, 30CST (times)			
Preoperative	6.57 ± 5.20 (4.6–8.5)	7.1 ± 1.88 (6.4–7.8)	0.59
Postoperative 6 months	10.10 ± 1.75 (9.4–10.7)	11.59 ± 2.30 (10.7–12.5)	0.09
Postoperative 12 months	11.1 ± 2.75 (10.1–12.1)	11.53 ± 1.98 (10.8–12.3)	0.49
40 m fast paced walk test, 40FPWT (m/sec)			
Preoperative	1.38 ± 0.27 (1.3–1.5)	1.44 ± 0.21 (1.4–1.5)	0.28
Postoperative 6 months	1.68 ± 0.21 (1.6–1.7)	1.71 ± 0.20 (1.6–1.8)	0.53
Postoperative 12 months	1.74 ± 0.19 (1.7–1.8)	1.80 ± 0.19 (1.7–1.9)	0.21
Timed up and go test, 3TUG (s)			
Preoperative	15.43 ± 2.99 (14.3–16.5)	14.74 ± 2.68 (13.7–15.7)	0.35
Postoperative 6 months	11.35 ± 1.59 (10.8–11.9)	10.61 ± 1.84 (9.9–11.3)	0.10
Postoperative 12 months	10.86 ± 1.71 (10.1–10.9)	10.14 ± 1.58 (9.6–10.7)	0.10

**Fig. 4:**
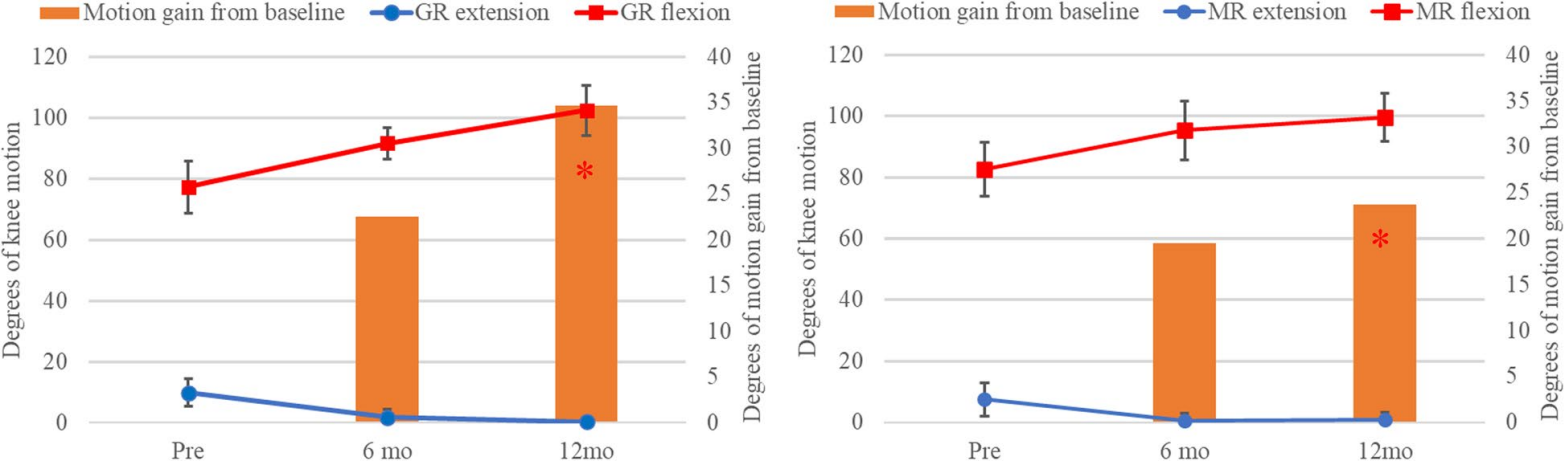
Showed degrees of knee motions and degrees of motion gain after 6 and 12 months postoperatively between GR and MR group

All patients in this trial had a genu varus and flexion contracture deformity with the HKA of 168° ± 5° and 168° ± 6° for GR and MR group (*p* = 0.8). There was no difference in post operative HKA between GR and MR group (176° ± 3° and 175° ± 3°, *p* = 0.38). Component positions between the two groups were not different as shown in Table 1. The mean baseline range of motion of GR group was 9.83°-77.17° (arc of motion 67.33° ± 10.56°), while the mean baseline range of motion of MR group was 7.5°-82.67° (arc of motion 75.17° ± 12.76°). At 6 months post TKA, a mean flexion angle of GR group was 91.5° ± 5.11° compared to 95.33° ± 9.55° for the MR group (*p* = 0.06). At 12 months post TKA, a mean flexion angle of GR group was 102.33° ± 8.17° compared to 99.67° ± 7.87° for the MR group (*p* = 0.2). Patients using GR design showed a greater improvement of knee motion after 6 months of TKA (22.5° ± 9.72° vs 19.5° ± 9.86°, *p* = 0.24); however, no statistical significance was detected. The change in degrees of knee motion after 12 months postoperatively was statistically significant greater for GR group compared to the MR group (34.67 ± 12.52 vs 23.67 ± 12.59, *p* = 0.001), Fig. 4.

## Discussion

Cruciate retaining (CR) knee prosthesis has gained more popularity over a decade reported by most national joint registrations. The drawback of CR TKA is unpredictably paradoxical femoral anterior translation [11]. There are three designs of femoral component based on the knee rotation center. First, the MR femoral prosthesis was designed using the transepicondylar axis as an axis for knee flexion and rotation. Second, the single radius (SR) femoral prothesis was proposed as a theory of the knee flexion–extension rotation center is located more distal and posterior to the transepicondylar axis, leading to a longer extensor moment arm, maintaining stability during knee motion due to equal ligament tension during knee flexion and extension, and thereby reducing the paradoxical anterior femoral movement. Lou et al. [12] reported the SR prosthesis had a greater knee motion (123° ± 10° vs 115° ± 10°) and less anterior knee pain (7% vs 18%) compared to the MR prosthesis. However, no difference in terms of clinical scales, radiographic result, satisfaction rates, and survival results was observed between MR and SR [13, 14]. Third, the GR femoral prosthesis was also designed using the transepicondylar axis with the point of rotation similar to the native knee. The philosophy of GR design is made to enhance motion, function, and mid-flexion stability by avoid the abrupt changes in the femoral sagittal radius curvature [7]. The in vitro study by Clary CW et al. [7] presented the magnitude of paradoxical anterior translation in mid flexion movement was decreased between 21 and 68% for the GR design. In vivo kinematic study showed the lessor paradoxical anterior slide was found with the GR design [15]. Pfitzner et al. also reported GR design decreased femorotibial translation compared with conventional design and improved impact of load and muscle force in unloaded and weight-bearing conditions [16].

However, the clinical outcome for GR design is still controversial. Ranawat et al. reported no difference outcome and radiographic between Attune implant (GR design) and PFC TKA (MR design) at 2-year follow up [17]. Carey BW et al. [18], on the contrast, found a significant difference in clinical outcome at 6 months postoperatively between Attune and PFC sigma. Etter K et al. [19] also showed GR design was better than conventional design in terms of length of stay, operative time, and discharge status. Among inconclusive evidence, this is the first study with level of evidence I to investigate the clinical significance between GR and MR design. Our study showed that both GR and MR designs presented comparable results in terms of patient reported outcome; KOOS, and performance-based tests; 30CST, 40FPWT, and 3TUG at 6 and 12 months postoperatively between GR and MR designs of CR TKA. All patients whether GR or MR designs could return their performance outcomes to normal which are 30CST > 10 times [17], 40FPWT > 1 m/sec [20], and 3TUG < 13.5 s [21]. Although the trial was intended to assess the non-inferiority of GR as compared with MR designed prosthesis in terms of functional outcomes after TKA, the findings demonstrated that the range of motion can gain significantly greater in the GR design compared to the MR design at 12 months postoperatively (34.67 ± 12.52 vs 23.67 ± 12.59, *p* = 0.001). The limitations for this trial were associated conditions which affect participants’ mobility such as contralateral knee pain and associated spinal deformity were not excluded.

## Conclusion

Both GR and MR cruciate retaining prostheses can lead to satisfactory outcomes at 6 and 12 months postoperatively. The GR prosthesis design gave more knee motions than did the MR prostheses. The GR prosthesis design is noninferior to the MR prosthesis design in terms of functional outcomes and performances.

## Data Availability

The datasets analyzed in this study are available from the corresponding author on reasonable request.
