# Correction: Clinical advantages of gradually reducing radius versus multi‑radius total knee arthroplasty: a noninferiority randomized trial

**DOI:** 10.1186/s12891-023-07017-1

**Published:** 2023-11-16

**Authors:** Sakkadech Limmahakhun, Anuchit Chaiamporn, Kasisin Klunklin, Warakorn Jingjit

**Affiliations:** Department of Orthopedic Surgery, Faculty of Medicine, Chiang Mai University, Chiang Mai, Thailand

**Correction:** ***BMC Musculoskelet Disord*** **24** **, 69 (2023)**

10.1186/s12891-023-06177-4

Following publication of the original article [1], the authors corrected the error on the date of the trial from “*conducted between January 2018—December 2020*“ to “*conducted in September 2020*“.

## Abstract section (method)

“This patient-blinded, parallel, non-inferiority trial conducted between January 2018—December 2020” has been changed to “This patient-blinded, parallel, non-inferiority trial conducted in September 2020”.

## Trial design section

“The trial was a single-center, CONSORT-compliant, patient-blinded, 2-group, parallel randomized controlled trial (RCT) conducted between January 2018—December 2020” has been changed to “The trial was a single-center, CONSORT-compliant, patient-blinded, 2-group, parallel randomized controlled trial (RCT) conducted in September 2020”.

The original article [1] has been updated.
